# 1345. Clinical Characteristics and Outcomes of Mild to Moderate COVID-19 Patients with Abnormal Chest Radiologic Findings Treated in the Hospitels in Thailand

**DOI:** 10.1093/ofid/ofad500.1182

**Published:** 2023-11-27

**Authors:** Naruemit Sayabovorn, Pochamana Phisalprapa, Weerachai Srivanichakorn, Thanet Chaisathaphol, Chaiwat Washirasaksiri, Tullaya Sitasuwan, Chonticha Auesomwang, Chayanis Kositamongkol, Pongpol Nimitpunya, Teerapat Attachitwatin, Methee Chayakulkeeree, Pakpoom Phoompoung, Cherdchai Nopmaneejumruslers, Tawatchai Taweemonkongsap, Visit Vamvanij, Rungsima Tinmanee

**Affiliations:** Faculty of Medicine Siriraj Hospital, Bangkok Noi, Krung Thep, Thailand; Faculty of Medicine Siriraj Hospital, Bangkok Noi, Krung Thep, Thailand; Faculty of Medicine Siriraj Hospital, Bangkok Noi, Krung Thep, Thailand; Faculty of Medicine Siriraj Hospital, Bangkok Noi, Krung Thep, Thailand; Faculty of Medicine Siriraj Hospital, Bangkok Noi, Krung Thep, Thailand; Faculty of Medicine Siriraj Hospital, Bangkok Noi, Krung Thep, Thailand; Faculty of Medicine Siriraj Hospital, Bangkok Noi, Krung Thep, Thailand; Faculty of Medicine Siriraj Hospital, Bangkok Noi, Krung Thep, Thailand; Faculty of Medicine Siriraj Hospital, Bangkok Noi, Krung Thep, Thailand; Faculty of Medicine Siriraj Hospital, Bangkok Noi, Krung Thep, Thailand; Faculty of Medicine Siriraj Hospital, Bangkok Noi, Krung Thep, Thailand; Faculty of Medicine Siriraj Hospital, Mahidol University, Bangkok, Krung Thep, Thailand; Faculty of Medicine Siriraj Hospital, Bangkok Noi, Krung Thep, Thailand; Faculty of Medicine Siriraj Hospital, Bangkok Noi, Krung Thep, Thailand; Faculty of Medicine Siriraj Hospital, Bangkok Noi, Krung Thep, Thailand; Faculty of Medicine Siriraj Hospital, Bangkok Noi, Krung Thep, Thailand

## Abstract

**Background:**

Hospitels are the hotels converted into the healthcare facilities extension in areas with limited resources including hospital beds during the peak of COVID-19 pandemic in Thailand. This study aimed to determine the clinical features and outcomes of asymptomatic and mild to moderate COVID-19 patients who were treated in these facilities.

**Methods:**

We conducted a retrospective study by reviewing the medical records of adult COVID-19 patients who were admitted to Siriraj Hospital’s affiliated hospitels in Bangkok between June and October 2021. Patients’ characteristics, vaccination status, treatment data, and clinical outcomes were collected.

**Results:**

We compared baseline characteristics and clinical features of COVID-19 patients based on their initial chest x-ray (CXR) findings. Of 1,729 patients, 644 (37.2%) had an abnormal baseline CXR, and these patients were older (49.2 vs. 42.2 years, *p* < 0.001), had higher body weight (68.1 vs. 64.7 kg, *p* < 0.001) and BMI (26.3 vs. 24.9 kg/m², *p* < 0.001), and were more likely to have underlying diseases such as diabetes (p = 0.002), hypertension (*p* < 0.001), obesity (*p* < 0.001), chronic lung disease (*p* = 0.029), and autoimmune disease (*p* = 0.028). They were also more likely to have a risk of disease progression (*p* < 0.001) and were less likely to be fully vaccinated (*p* < 0.001). They were more likely to be referred back to the hospital (*p* < 0.001). We found no significant differences in sex or COVID-19 symptoms between the two groups, except cough and fatigue. The total cost of admission was significantly higher for referred patients than discharged patients (70,545 vs. 12,010 Thai baht, *p* < 0.001).

**Conclusion:**

A significant proportion of COVID-19 patients with abnormal CXR findings who were admitted to the hospitels had underlying diseases and higher risk of disease progression. Additionally, they were more likely to be referred back to the hospital and had higher healthcare costs. Continued surveillance and monitoring of high-risk COVID-19 patients in hospitels to ensure efficient management and resource utilization were needed.

**Disclosures:**

**All Authors**: No reported disclosures

